# Intergenerational enrollment and expenditure changes in Medicaid: trends from 1991 to 2005

**DOI:** 10.1186/1472-6963-12-327

**Published:** 2012-09-20

**Authors:** Stephen W Patrick, Gary L Freed

**Affiliations:** 1 The Department of Pediatrics and Communicable Diseases, University of Michigan Health System, Ann Arbor, MI, 48109, USA; 2 Robert Wood Johnson Foundation Clinical Scholars Program, University of Michigan Health System, Ann Arbor, MI, 48109, USA; 3 Division of Neonatal-Perinatal Medicine, University of Michigan Health System, Ann Arbor, MI, 48109, USA; 4 The Department of Pediatrics and Communicable Diseases, Child Health Evaluation and Research Unit, University of Michigan Health System, Ann Arbor, MI, 48109, USA; 5 6312 Medical Science Building 1, 1150 W. Medical Center Drive SPC 5604, Ann Arbor, MI, 48109-5604, USA

**Keywords:** Medicaid, CHIP, Long-term care, Disabled

## Abstract

**Background:**

From its inception, Medicaid was aimed at providing insurance coverage for low income children, elderly, and disabled. Since this time, children have become a smaller proportion of the US population and Medicaid has expanded to additional eligibility groups. We sought to evaluate relative growth in spending in the Medicaid program between children and adults from 1991-2005. We hypothesize that this shifting demographic will result in fewer resources being allocated to children in the Medicaid program.

**Methods:**

We utilized retrospective enrollment and expenditure data for children, adults and the elderly from 1991 to 2005 for both Medicaid and Children’s Health Insurance Program Medicaid expansion programs. Data were obtained from the Centers for Medicare and Medicaid Services using their Medicaid Statistical Information System.

**Results:**

From 1991 to 2005, the number of enrollees increased by 83% to 58.7 million. This includes increases of 33% for children, 100% for adults and 50% for the elderly. Concurrently, total expenditures nationwide rose 150% to $273 billion. Expenditures for children increased from $23.4 to $65.7 billion, adults from $46.2 to $123.6 billion, and elderly from $39.2 to $71.3 billion. From 1999 to 2005, Medicaid spending on long-term care increased by 31% to $84.3 billion. Expenditures on the disabled grew by 61% to $119 billion. In total, the disabled account for 43% and long-term care 31%, of the total Medicaid budget.

**Conclusion:**

Our study did not find an absolute decrease in the overall resources being directed toward children. However, increased spending on adults on a per-capita and absolute basis, particularly disabled adults, is responsible for much of the growth in spending over the past 15 years. Medicaid expenditures have grown faster than inflation and overall national health expenditures. A national strategy is needed to ensure adequate coverage for Medicaid recipients while dealing with the ongoing constraints of state and federal budgets.

## Background

When Medicaid, Title XIX of the Social Security Act, began in 1965, it was aimed at providing insurance coverage for low income children, elderly, and disabled. Medicaid, an entitlement program, relies on a unique federal-state partnership for financing and is administered at the state level. Because of this, Medicaid has been able to “formulate creative structural solutions and implement reforms” [1] tailored to state needs. Through the years the program has seen incremental expansions in covering a greater proportion of poor children and pregnant women. Medicaid has also expanded coverage beyond what could have been conceptualized in 1965 to include such things as coverage for breast cancer and AIDS treatment. Furthermore, all states now cover many “optional” services -including prescription drugs and long-term care.

The Children’s Health Insurance Program (CHIP), Title XXI of the Social Security Act, began in 1998 and aimed to provide health insurance coverage to low income, uninsured children not eligible for Medicaid. Unlike Medicaid, CHIP is a block grant, not an entitlement program. As with Medicaid, CHIP is financed with both federal and state dollars and is administered at the state level. In administering the program, states are given the option of having CHIP combined with their Medicaid program, as a stand-alone program or as an expansion to their Medicaid program. States that choose to have a Medicaid expansion CHIP program must meet the same mandated benefit packages as Medicaid [2].

Historically, because of Medicaid and CHIP’s partial dependence upon state general revenues, when state revenues dropped and healthcare costs rose, programs were often cut or curtailed [1]. During harsh economic times earlier this decade, states were faced with growing enrollment, medical inflation and plummeting state revenues [3]. In response, many states implemented barriers to secure and retain coverage in their Medicaid and CHIP programs [4]. Recently, as the U.S. economy has faltered, enrollment has increased, medical inflation has outpaced overall inflation [5] and state budgets are again in crisis.

While Medicaid has continued to expand in scope, one of the program’s original target populations, children, have become a smaller proportion of the United States population. In 1966 those under age 18 accounted for 37 percent of the total population [6]; whereas, in 2005 they accounted for just 25 percent [7]. It has been speculated that as America ages, resources will be preferentially allocated to the growing elderly population [8]. Previous studies have evaluated total United States social spending and found that spending per capita for children, particularly in challenging economic times, has not kept pace with per capita spending for the elderly [9,10]. We hypothesize that this shifting demographic will result in fewer resources being allocated to children in the Medicaid program.

## Methods

Data from all 50 states were obtained from the Centers for Medicare and Medicaid Services (CMS) using their Medicaid Statistical Information System (MSIS). Prior to 1999, data were collected using the HCFA-2082 hard-copy reporting process. Beginning with FY 1999, the Balanced Budget Act (BBA) of 1997 requires states to submit all eligibility and claims data to CMS on a quarterly basis through the MSIS. Because of this reporting change, data after 1999 were available to examine specific age groups in specific service categories such as long-term care. We evaluated data from the MSIS from 1991 to 2005, looking at trends in enrollment and expenditures focusing on children, adults and the elderly. Because of the constraints of the data sources, for analyses which include data from 1991 to 1998, children are considered aged birth to 20 years and adults are 21 to 64 years; whereas, for analyses which includes data only after 1999 children are considered birth to 18 years and adults 19 to 64 years. Throughout all analyses, the elderly are always categorized as aged 65 years and greater. For each group we evaluated their expenditures for long-term care, and also enrollment and expenditures for those whose basis for eligibility in the Medicaid program was due to being classified as “blind/disabled.” Long-term care includes expenditures on Home Health Services, Intermediate Care Facility Services for the Mentally Retarded, Mental Health Facility Services and Personal Support Services. All findings were adjusted for inflation based upon the year 2005, unless otherwise specified. For simplicity, we refer to both Medicaid and Medicaid Expansion CHIP programs as “Medicaid.”

The data do not include Disproportionate Share Hospital (DSH) Payments and only include enrollment and expenditures for Medicaid Expansion CHIP. The category “adjustments” are lump sum payments made for Medicaid enrollees. These payments include add-on programs including those for inpatient, outpatient, psychiatric, pediatric, critical care and Medicaid high-volume providers. An example would be the Illinois Rural Critical Hospital Adjustment Payment Process, which is a quarterly payment program that provides rural Illinois hospitals with additional Medicaid payments based upon either their obstetrical care or general care admissions from a pre-determined base period [10]. Because all data were aggregate, de-identified data obtained from public sources, this study was exempt from human subjects consideration.

## Results

### Trends in Medicaid enrollment

**Figure 1:**
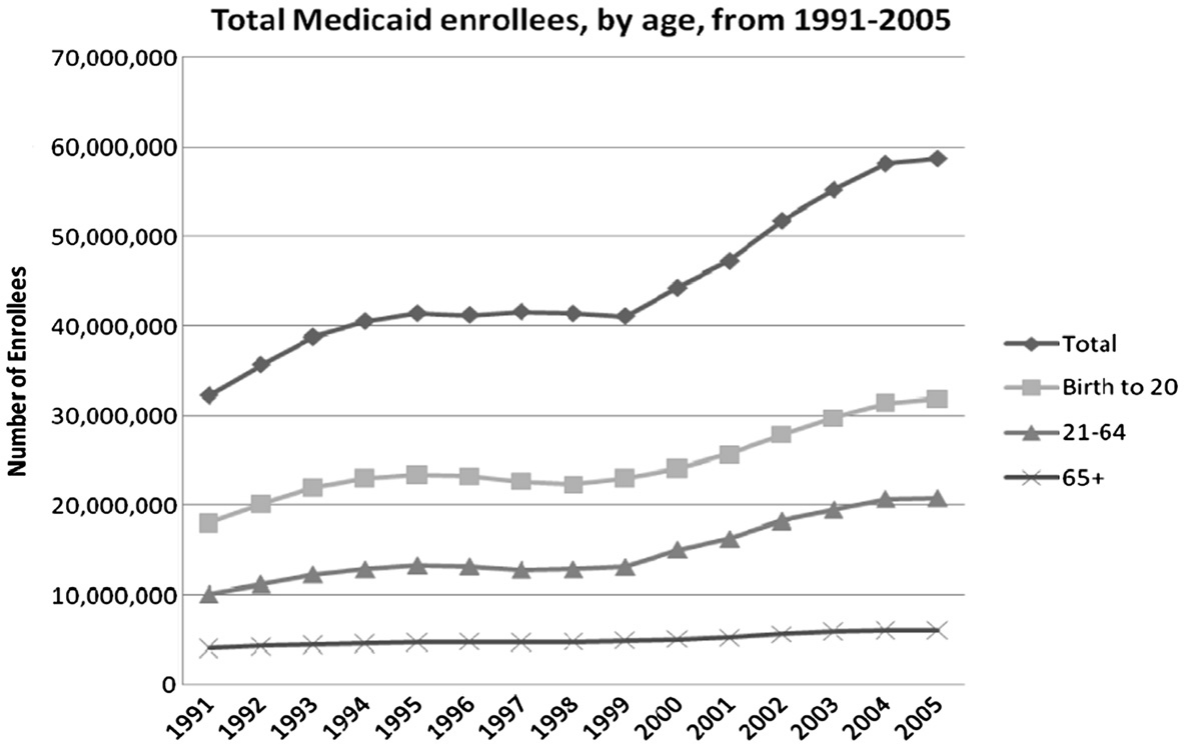
Total Medicaid enrollees, by age, from 1991-2005.

From 1991 to 2005, the absolute number of Medicaid enrollees nearly doubled from 32.2 to 58.7 million. Children increased by more than 33 percent to 31.8 million, adults increased by 100 percent to 20.7 million, and the elderly increased by approximately 50 percent from 4 to 6 million (Figure 1).

Overall, children as a proportion of total Medicaid enrollment held steady at approximately 55 percent. Enrollment for non-elderly adults increased from 31.3 percent to 35.4 percent of all beneficiaries, and the proportion of elderly decreased from 12.5 percent to 10.3 percent.

### Trends in Medicaid expenditures

**Figure 2:**
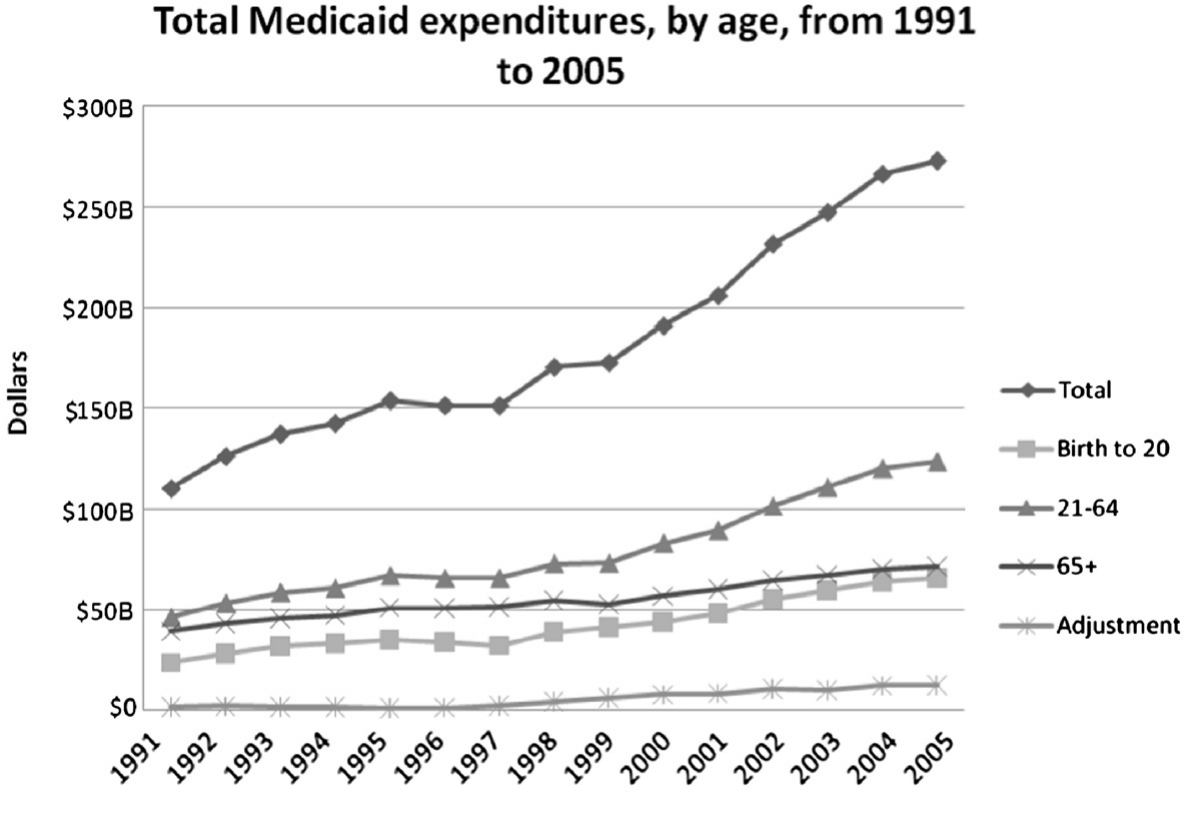
Total Medicaid expenditures, by age, from 1991 to 2005.

Over the same time period, total annual Medicaid expenditures nationwide rose from $110 to $273 billion, an increase of almost 150 percent. This includes increases in expenditures for children from $23.4 to $65.7 billion, adults from $46.2 to $123.6 billion, and elderly from $39.2 to $71.3 billion (Figure 2).

**Figure 3:**
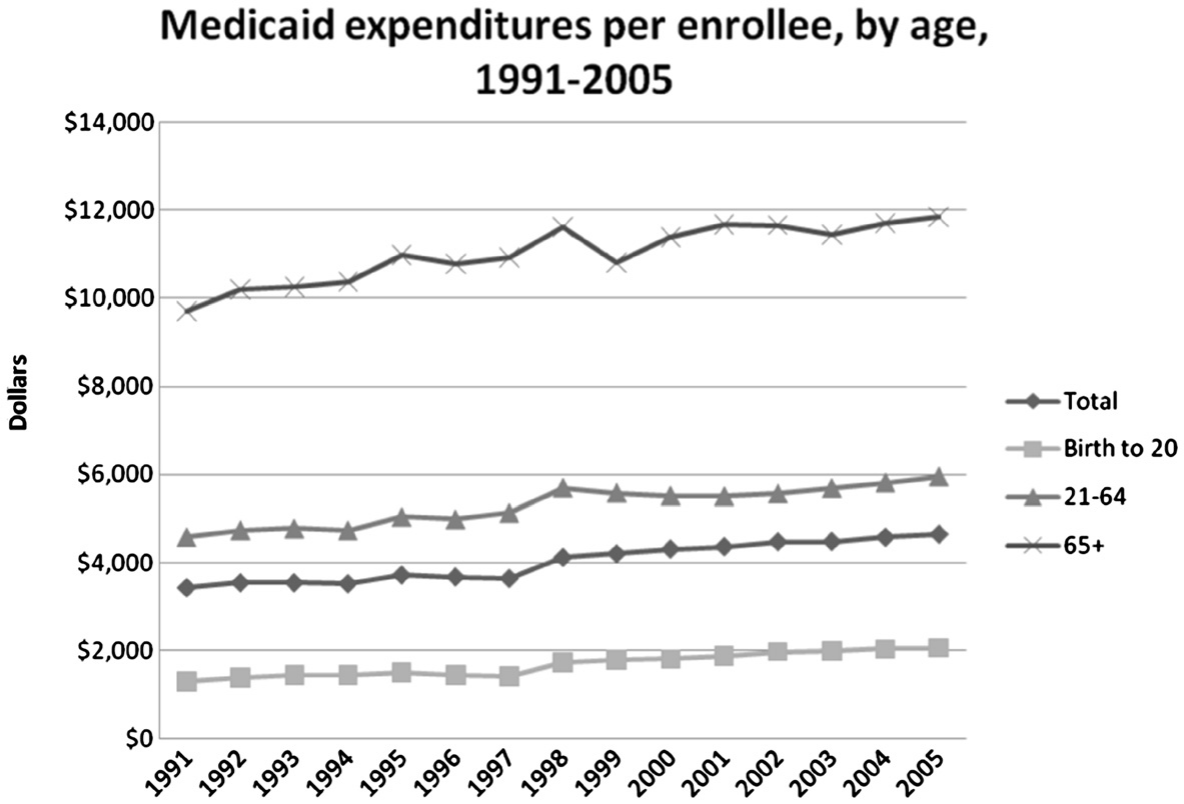
Medicaid expenditures per enrollee, by age, 1991-2005.

From 1991 to 2005, there were increased per capita expenditures across all groups, averaging increases of $763 for children, $1,366 for adults and $2,133 for the elderly. Average cost per enrollee in the entire program increased by $1,219, with the highest cost per enrollee for the elderly and the lowest for children (Figure 3).

Since 1991, expenditures for children and adults as a proportion of overall Medicaid expenditures increased from 21.2 to 24.1 percent and 41.8 to 45.3 percent, respectively; whereas, the overall proportion of expenditures for the elderly decreased from 35.5 percent to 26.1 percent.

From 1991 to 2005, children accounted for 52.2 percent of growth in enrollment and 26.0 percent of growth in expenditures. This is in contrast to adults who accounted for 40.3 percent of enrollment growth and 47.6 percent of growth in expenditures, and the elderly which accounted for 7.5 percent enrollment growth and 19.7 percent expenditures growth (Table 1).

**Table 1:** Growth in Enrollment and Expenditures by Age, 1991-2005

	**Enrollment**	**Expenditures**
Children	52.2%	26.0%
Adults	40.3%	47.6%
Elderly	7.5%	19.7%

### Long-term care

Long-term care remains a major proportion of the overall Medicaid budget. In 1999, long-term care accounted for 37.3 percent of total Medicaid expenditures, decreasing to 30.9 percent in 2005. For the elderly, long-term care continues to account for the majority of their Medicaid expenditures – 70 percent in 1999 and 62.9 percent in 2005. Expenditures on long-term care have steadily increased, but have not grown as rapidly as overall Medicaid expenditures.

**Figure 4:**
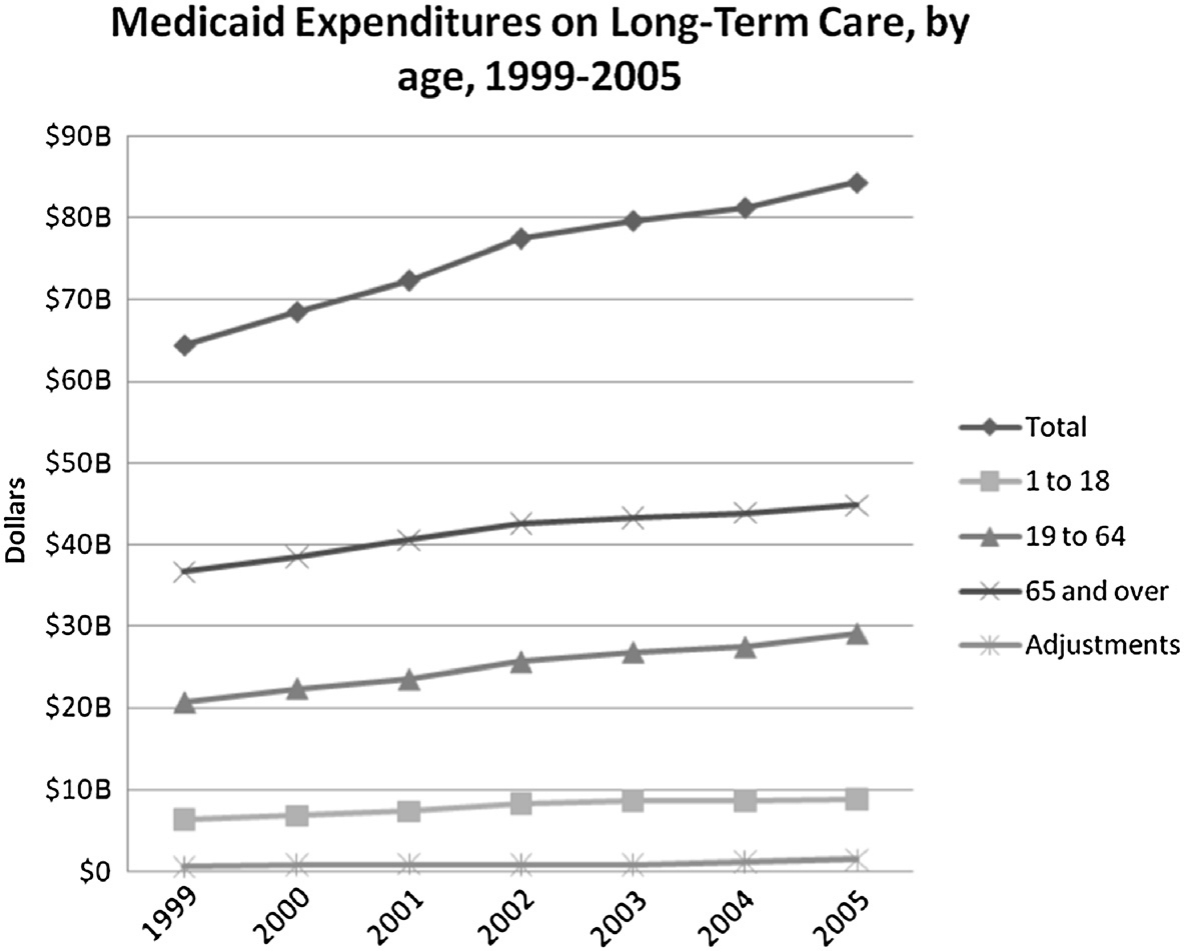
**Medicaid Expenditures on Long-Term Care, by age, 1999-2005.** (Long-Term Care includes expenditures on Home Health Services, Intermediate Care Facility Services for the Mentally Retarded, Mental health Facility Services and Personal Support Services).

From 1999 to 2005, Medicaid spending on long-term care increased by 31 percent or $19.9 billion; from $64.4 to $84.3 billion. This increase was consistent in all age groups, with an increase for children of $2.5 billion, from $6.4 to $8.9 billion, for adults of $8.4 billion, from $20.7 to $29.1 billion, and for the elderly of $10 billion, from $36.7 to $44.9 billion (Figure 4).

### Disabled

The majority of those whose basis of eligibility for Medicaid is “disabled” have been, and continue to be, aged 19-64 years. The number of disabled adults enrolled in Medicaid grew steadily from 1999 to 2005, while the numbers of disabled children and the elderly did not change markedly. From 1999 to 2005, adult disabled enrollees increased from 5.4 million to 6.8 million, while the number of disabled children on Medicaid grew from 1.25 million to 1.3 million and the elderly from 641,000 to 699,000.

**Figure 5:**
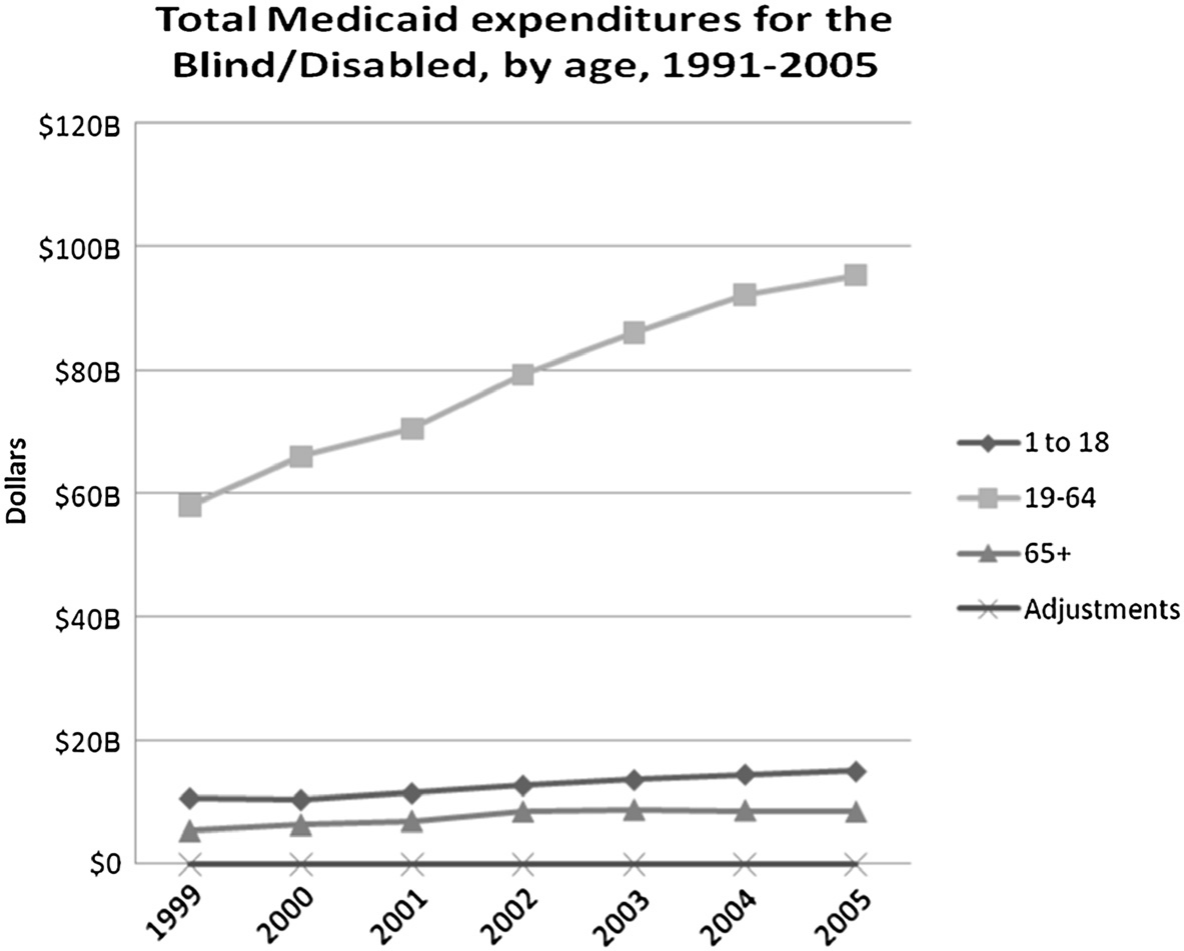
Total Medicaid expenditures for the Blind/Disabled, by age, 1991-2005.

Disabled adults continue to be the most costly among the disabled, with expenditures increasing from $58 billion in 1999 to $95.3 billion in 2005. In 2005, this group alone accounted for greater than 35 percent of total Medicaid expenditures. Disabled children and elderly experienced smaller increases from 1999 to 2005 - disabled children increasing from $10.6 to $15 billion and disabled elderly increasing from $5.3 to $8.4 billion (Figure 5).

## Discussion

Without adjusting for inflation, from 1991 to 2005, Medicaid expenditures grew by almost 250 percent. By comparison, over the same period, national health expenditures grew by approximately 165 percent and overall inflation increased by 43 percent [5,11,12]. We hypothesized that the decreasing proportion of children in the United States would result in decreased resources being allocated to children; however, this did not occur during our study period. It is likely that child enrollment and expenditures in Medicaid were bolstered by new programs targeted at children, such as the Children’s Health Insurance Program. Our study does find, however, that most of the new spending in the program has been targeted toward adults. We find that expenditures for adults in Medicaid are growing faster than children or the elderly.

### The disabled

Although the disabled account for fifteen percent of Medicaid enrollees, they account for over forty percent of total Medicaid expenditures - disabled adults alone now accounting for over a third of total Medicaid expenditures. The absolute number of disabled beneficiaries has grown for several reasons. While states have borne the responsibility of caring for the disabled through the years, their emergence in the Medicaid program has been more recent. Over the last several decades, care for the disabled has been gradually deinstitutionalized and increasingly “medicalized” through an effort to “convert service programs for disabled children, developmentally disabled and the chronically ill to Medicaid-based financing” [13]. In addition, since Medicaid’s enactment, there have been incremental expansions in eligibility, and the group now encompasses “disabled children, physically disabled but cognitively intact nonelderly adults, the developmentally disabled and people with severe and persistent mental illness,” and low-income people with AIDS [13]. Lastly, there have been greatly reduced mortality rates among certain categories of disabled adults, resulting in longer life spans. Enrollment and expenditures for the disabled are likely to increase as medical technology continues to improve, extending life for those who would not have survived even just a few years ago. Thus, proportional increases in spending for disabled adults are likely to continue.

### Long-term care

Medicaid continues to be the largest purchaser of long-term care in the United States [14] and this category accounts for nearly one-third of total Medicaid expenditures. A significant proportion of Medicaid enrollees that require long-term care are also enrolled in Medicare – termed “dual-eligible beneficiaries.” Medicaid assists these low income Medicare beneficiaries by paying Medicare premiums and providing Medicare uncovered services such as long-term care [15]. Expenditures on long-term care rose faster than inflation, but not as fast as total Medicaid expenditures. This is because of a trend towards community-based instead of institution based service delivery [13]. More emphasis has been placed on community-based treatment after the Supreme Court decision of June of 1999 in the case of Olmstead vs. LC, which requires states to provide community-based treatment where appropriate [16].

### State effects

The strain of the rapid growth of the Medicaid program is clearly felt by the states. In 1991, elementary and secondary education accounted for an average of 22.4 percent of state budgets compared to 13.6 percent for Medicaid [17]. By 2007, Medicaid tied elementary and secondary education as the most expensive line item in state budgets, accounting for an average of 21.2 percent of the total; nine states reported spending more than a quarter of their state budget on their Medicaid program, with one state, Missouri, spending 35 percent [18].

States adopted several strategies to contain costs, including: changes in income verification requirements and enrollment procedures [4], changes to eligibility rules, changes in eligibility standards (such as reducing coverage for parents) provider payment rate reductions or freezes, increased controls on pharmacy utilization, optional benefit elimination for adults, increasing premiums and copayments where allowed, increasing managed care (including automatically assigning enrollees), instituting disease and case management programs, reducing payments to long-term care facilities, and investigations into fraud and abuse [19]. Given the broad nature of these cost-containment measures employed earlier this decade, there is concern that states are left with few opportunities for further cost savings [20].

### Future implications

#### Health reform

A key component of the Patient Protection and Affordable Care Act includes expansion of Medicaid eligibility to provide coverage for all Americans up to 133 percent of the federal poverty level [21]. This will result in millions of new enrollees to the Medicaid program. These enrollees, particularly childless adults, are not currently eligible in many states. To encourage states to enrollee newly eligible populations, beginning in 2014, the federal government will bear 100 percent of the costs of these new enrollees, decreasing to 90 percent in 2020. Governors, particularly in states with high levels of poverty, are expressing concern that such an expansion could financially cripple states [22].

### Limitations

Our study does have some clear limitations. First, data from MSIS is reported from states to the federal government. In the process of reporting, both errors of omission and commission are possible. Additionally, while we aggregate all states together to make inferences at the national level, this approach could overlook effects at the state level.

## Conclusions

By 2005, Medicaid provided health insurance coverage more than a quarter of US children and nearly one in ten US adults and elderly [23]. Growth in the Medicaid program outpaces both inflation and national health expenditures. In our current economic climate, where both budgetary cuts and eligibility expansions are being proposed, it is fair to ask where the breaking point of the Medicaid program might be. If Medicaid continues to grow at this pace it will continue to be a target for state and federal legislators looking to reduce overall state expenditures. The challenge for our nation’s future will be how to provide needed health care to each of Medicaid’s target populations within the constraints of the current US economy. We believe providing health care to our nation’s safety net, including our children, must continue to be a national priority. Innovative financing and structural changes to Medicaid which would ensure access to health care while being fiscally prudent must be a research priority for health services researchers in the coming years.

## Abbreviations

(CHIP): Children’s health insurance program; (CMS): Centers for medicare and Medicaid services; (MSIS): Medicaid statistical information system; (BBA): Balanced budget act; (DSH): Disproportionate share hospital; (AIDS): Acquired immune deficiency syndrome.

## Competing interests

Both authors declare that they have no competing interests.

## Authors’ contribution

All authors (SP, GF) contributed to the analysis, interpretations of the data, drafting and revision of the manuscript. All authors approved the final manuscript.

## Conflicts

The authors have no conflicts of interests or corporate sponsors to disclose.

## Funding source

The authors would like to thank the Robert Wood Johnson Foundation for their support of this work.

## Support

The Robert Wood Johnson Foundation Clinical Scholars Program provided support for this project.

## Pre-publication history

The pre-publication history for this paper can be accessed here:

http://www.biomedcentral.com/1472-6963/12/327/prepub
